# Coexistence of Mosaic Uniparental Isodisomy and a *KCNJ11* Mutation Presenting as Diffuse Congenital Hyperinsulinism and Hemihypertrophy

**DOI:** 10.1159/000446153

**Published:** 2016-05-14

**Authors:** Pınar Kocaay, Zeynep Şiklar, Sian Ellard, Aydın Yagmurlu, Emine Çamtosun, Esra Erden, Merih Berberoglu, Sarah E. Flanagan

**Affiliations:** ^a^Department of Pediatric Endocrinology, Ankara University Faculty of Medicine, Ankara, Turkey; ^b^Department of Pediatric Surgery, Ankara University Faculty of Medicine, Ankara, Turkey; ^c^Department of Pathology, Ankara University Faculty of Medicine, Ankara, Turkey; ^d^Institute of Biomedicaland Clinical Science, University of Exeter Medical School, Exeter, UK

**Keywords:** Congenital hyperinsulinism, Uniparental isodisomy, *KCNJ11*, Beckwith-Wiedemann syndrome, Hypoglycaemia

## Abstract

**Background:**

Isolated hyperinsulinaemic hypoglycaemia (HH) commonly results from recessively inherited mutations in the *ABCC8* and *KCNJ11* genes that are located on chromosome 11p15.1. More rarely, HH can feature in patients with Beckwith-Wiedemann syndrome (BWS), a congenital overgrowth disorder, resulting from defects at a differentially methylated region telomeric to the K-ATP channel genes at chromosome 11p15.5.

**Subject:**

We undertook genetic testing in a patient with diazoxide-unresponsive HH diagnosed at birth. Physical examination later revealed hemihypertrophy of the right arm, a feature of BWS.

**Results:**

We identified a novel mosaic, paternally-inherited *KCNJ11* mutation(s) in the patient. Further analysis confirmed uniparental disomy (UPD) of chromosome 11, which extended across the *KCNJ11* gene at 11p15.1 and the BWS locus at 11p15.5.

**Conclusion:**

These results highlight the importance of considering UPD as a mechanism of disease in patients with HH and a paternally inherited K-ATP channel mutation, especially when additional syndromic features are present.

## Established Facts

- Rarely, hyperinsulinaemic hypoglycaemia can present as part of Beckwith-Wiedemann-syndrome (BWS), a severe overgrowth disorder characterized by macroglossia, abdominal wall defects, hemihypertrophy, macrosomia, hypoglycaemia and an increased risk of tumors.
- Focal hyperinsulinism where uniparental disomy (UPD) of chromosomes 11p15.5-11p15.1 occurs in a single pancreatic cell. This unmasks a paternally inherited K-ATP channel mutation and provides the cell with a selective growth advantage due to the inappropriate expression of imprinted genes at 11p15.5, leading to clonal expansion.

## Novel Insights

- BWS should be considered in patients with newly diagnosed hyperinsulinaemic hyperglycaemia even in the absence of additional clinical features.
- UPD should also be considered in all patients with persistent hyperinsulinism with a paternally inherited, recessively acting K-ATP channel mutation even in the absence of focal pancreatic disease. A mitotic recombination event, which led to the UPD of chromosome 11, may have occurred earlier and most likely prior to the differentiation of the pancreatic precursor cells.

## Introduction

Hyperinsulinaemic hypoglycaemia (HH) is characterized by the unregulated secretion of insulin despite low blood glucose and is the major cause of persistent hypoglycaemia in the newborn and infancy periods [1]. A rapid diagnosis and appropriate treatment is essential in order to prevent brain injury. A range in the clinical severity of disease is observed, with some patients having asymptomatic hypoglycaemia, whilst others present with severe, medically unresponsive disease that requires pancreatectomy.

Heterogeneity is also observed with regard to the underlying genetic aetiology with mutations in 9 different genes reported [2]. Of these, the commonest genetic aetiology is loss-of-function mutation(s) in the *ABCC8* and *KCNJ11* genes, which encode the sulphonylurea receptor 1 and inward-rectifying potassium (Kir6.2) subunits of the pancreatic ATP-sensitive potassium (K-ATP) channel. Mutations in these two genes account for 45% of all HH cases and are important to identify as the mode of inheritance of mutations provides information that can guide clinical management. Histologically, two forms of the disease exist; diffuse and focal. Focal lesions develop when paternal uniparental disomy (UPD) of chromosome 11p15 occurs in a single cell within the developing pancreas. This results in an imbalance of imprinted genes involved in cell cycle regulation at 11p15.5, resulting in clonal expansion of the cell. The UPD also unmasks a recessively acting, paternally-inherited K-ATP channel mutation at 11p15.1, resulting in unregulated insulin release from the focal lesion [3]. Surgical removal of the focal lesion is usually curative. In contrast, diffuse disease affects the entire pancreas and most commonly results from the inheritance of two recessive mutations [2]. These mutations often lead to severe, medically unresponsive HH, which may require near total pancreatectomy. The lack of response to diazoxide, a K-ATP channel opener, in these patients can be explained by the absence of functional K-ATP channels at the cell membrane.

Rarely, HH can present as part of a syndrome, the most common being Beckwith-Wiedemann Syndrome (BWS), a severe overgrowth disorder characterized by macroglossia, abdominal wall defects, hemihypertrophy, macrosomia, hypoglycaemia and increased risk of tumors. A number of different genetic mechanisms can lead to BWS, all of which result in abnormalities in methylation at one of two imprinting centers (ICR1 and ICR2) on chromosome 11p15.5 [4]. In approximately 20-30% of the cases, BWS results from paternal UPD across the 11p15.5 region, leading to an imbalance in imprinting and dysregulation of genes that are important for cell cycle regulation [4]. As UPD is a sporadic event, which may occur during embryogenesis, there is often variability in the tissues that are affected. This can explain the differences in phenotype observed between individuals with BWS.

Recently, a few patients with BWS and persistent HH due to paternal UPD of chromosome 11, which extends from the BWS locus at 11p15.5 to the K-ATP channel genes at 11p15.1, have been reported. In these patients, the persistent hyperinsulinism results from the unmasking of a recessively inherited mutation in *ABCC8* or *KCNJ11* within the pancreatic tissue [5,6]. We now report a patient with congenital hyperinsulinism with hemihypertrophy resulting from novel paternally inherited, recessively acting *KCNJ11* mutation(s), which have been unmasked by paternal uniparental isodisomy that extends from 11p15.5 to 11p15.1 within the pancreatic tissue.

## Methods

### Genetics

Genomic DNA was extracted from peripheral leukocytes and formalin-fixed, paraffin-embedded pancreatic tissue, using standard procedures. The coding regions and intron/exon boundaries of the *KCNJ11* and *ABCC8* genes were amplified by PCR using DNA extracted from leukocytes. The PCR products were Sanger sequenced, and the traces were compared to published sequences using Mutation Surveyor Software.

Seven microsatellite markers spanning chromosomes 11p15.5-11p15.1 were amplified in DNA extracted from the formalin-fixed, paraffin-embedded pancreatic tissue and leukocyte DNA from the patient and both parents. The resulting data were analysed using Genemarker software (Soft Genetics, State College, Pa., USA).

## Results

### Clinical Characteristics

The female patient was macrosomic at birth (3.8 kg at 35 weeks' gestation) and developed refractory hypoglycaemia immediately after birth. Laboratory investigations revealed HH [blood glucose: 22 mg/dl (1.2 mmol/l); paired insulin: 52.6 mIU/ml; ketones: <0.2 mmol/l, and cortisol: 14.4 mg/dl (normal range: 6.2-19.4)]. Glucose infusion at a rate of 20 mg/kg/min was required to maintain euglycaemia. Diazoxide was introduced at a dose of 10 mg/kg/day and was increased to 20 mg/kg/day without effect. Octreotide was subsequently added to the treatment regimen at a maximum dose of 40 mg/kg/day, which did not result in a resolution of hypoglycaemia. Due to the poor response to medical therapy, the patient underwent a near total pancreatectomy at the age of 10 weeks. Histological analysis of the resected pancreatic tissue demonstrated diffuse islet cell hypertrophy and hyperplasia. Post-operative hypoglycaemia was observed, and octreotide (10 μg/kg/day) was reintroduced, which resulted in euglycaemia.

At the age of 8 weeks, a 3/6 systolic murmur was detected in the aortic focus. Physical examination identified macrosomia [height: 57 cm (-0.37 SDS), body weight: 5,180 g, respondent BMI: 98.91%, head circumference: 37 cm] and a 1.5-cm difference in circumference between the left and right arm. A diagnosis of hemihypertrophy of the right arm was recorded. At the age of 3 years, and due to suspicion of non-compliance, treatment with a long-acting sandostatin analog was introduced to manage recurring hypoglycaemia.

The patient is currently 3 years and 2 months of age and continues to have monthly injections of long-acting octreotide without episodes of hypoglycaemia (current HbA1c 5.2%). The patient has a respondent BMI of 91.76% [weight 14 kg, height 100 cm (0.1 SDS)] and is meeting all of her developmental milestones. Hemihypertrophy of the right arm is still evident. An electrocardiogram was performed following the detection of the heart murmur. A pulmonary valvular stenosis and patent ductus arteriosus were detected, both of which have now resolved without the need for medical intervention. An abdominal ultrasound scan was undertaken, and no abnormalities were detected. Tumour markers have also been measured (alpha fetoprotein, beta human chorionic gonadotrophin and chorionic embryonic antigen, and all were within the normal range.

### Genetic Analysis

**Fig. 1:**
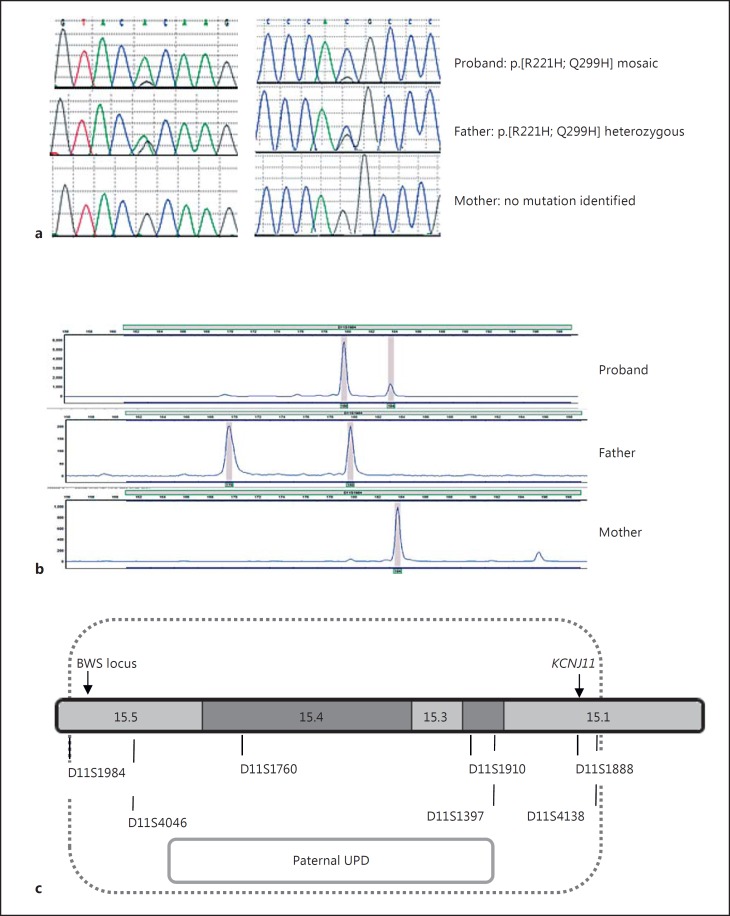
**a** Sequence electropherograms showing the p.R221H (c.662G>A; left panel) and p.Q299H (c.879G>C; right panel) *KCNJ11* mutation(s). The proband is mosaic for both mutations, whilst the father is heterozygous. Neither mutation was found in the sample from the mother. **b** Electropherograms demonstrating the results of microsatellite analysis of the informative marker (D11S1984) in the proband (pancreatic tissue) and her parents (leukocytes). Data for the additional 6 informative markers are not shown. The x-axis indicates the product size [base pairs (bp)], and the y-axis the product quantity (arbitrary units). The results illustrate mosaic UPD with a larger peak for the paternal allele (180 bp) compared with the maternal allele (184 bp). **c** Results of chromosome 11 microsatellite analysis for DNA extracted from the proband (pancreatic tissue) and her parents (leukocytes). The 7 informative markers, which demonstrated paternal UPD, are shown along with their approximate location on chromosome 11. The position of *KCNJ11* and the differentially methylated BWS locus are provided.

Sequence analysis identified two novel missense mutations, p.R221H (c.662G>A) and p.Q299H (c.897G>C), in the patient's *KCNJ11* gene. Both variants affect residues that are highly conserved across species, and in silico analysis is consistent with one or both of the mutations being disease causing (Alamut Rouen, France). Close inspection of the sequencing electropherogram showed that both variants were present at approximately 90% in the patient's leukocyte DNA sample. Testing of the parental samples confirmed that the unaffected father was heterozygous for the two variants, whilst neither mutation was found in the maternal sample (fig. 1a). Microsatellite analysis of markers on chromosome 11 confirmed that the patient had mosaic paternal UPD of chromosome 11 spanning a minimum region of 16.2 Mb, which encompassed the differentially methylated region at 11p15.5 (the BWS locus) and the *KCNJ11* gene at 11p15.1 in the pancreatic and leukocyte DNA (fig. 1b, c).

## Discussion

We report a patient with congenital HH and hemihypertrophy with two novel, paternally inherited *KCNJ11* mutations, which have been unmasked by mosaic paternal uniparental isodisomy within the pancreas. The father who is heterozygous for both mutations is clinically unaffected, consistent with the mutation(s) being recessively acting. In our patient, it is likely that the persistent hyperinsulinism was due to a loss of functional K-ATP channels within the pancreatic beta cell as a result of the *KCNJ11* mutation(s) [2]. This is further supported by the patient's lack of response to the drug diazoxide, which works by binding to and opening functional K-ATP channels, thereby preventing insulin secretion. However, functional studies will be required to confirm whether both or just one of the novel mutations is pathogenic.

Whilst the majority of cases with paternally inherited K-ATP channel mutations have a focal pancreatic lesion, our patient had diffuse pancreatic disease. The identification of mosaic UPD within the leukocyte DNA prompted us to consider the possibility of diffuse disease, which was later confirmed by histological analysis. This finding highlights the importance of performing imaging studies in all individuals with paternally inherited K-ATP channel mutations and medically unresponsive disease prior to surgery. The level of mosaicism observed within the pancreatic and leukocyte DNA in our patient was high (90%). This suggests that the mitotic recombination event, which led to UPD of chromosome 11, occurred early during development in our patient and most likely prior to the differentiation of the pancreatic precursor cells, thus resulting in all, or at least the majority of the cells, within the pancreas being affected. This is in contrast to focal hyperinsulinism, where UPD of chromosomes 11p15.5-11p15.1 is not detected in the blood as the genetic event occurs in a single pancreatic cell resulting in a selective growth advantage and clonal expansion [3].

The absence of additional features of BWS in our patient such as macroglossia, ear pit creases and abdominal wall defects suggests variability in the level of mosaicism or differences in mutation thresholds within different organs. Nephroblastomas and hepatoblastomas have been reported to develop in up to 24% of the individuals with BWS resulting from UPD of chromosome 11p15, with the risk being dependent on the level of mosaicism for UPD within each organ [4,6]. As a high level of mosaicism was detected in our patient, suggesting an early mutational event, it does seem likely that other tissues will be affected. As other tissues from our patient were not available for testing, it is not known which other organs are affected by UPD of chromosome 11. Ultrasonographic imaging and laboratory assessments will be performed annually to monitor for tumor development. The variability of mosaicism observed between tissues in patients with BWS also highlights the importance of the source of DNA for genetic studies. For example, in our patient, a low level or absence of mosaicism within the leukocytes may have resulted in the UPD being ‘missed’ by Sanger sequencing. This is particularly important to remember for patients with diffuse disease where pancreatic tissue is not available for testing, but where a paternally inherited K-ATP channel mutation has been identified.

Recently, mosaic UPD of chromosomes 11p15.5-11p15.1, resulting in the unmasking of a recessively acting K-ATP channel mutation, has been reported in 26 patients with BWS and persistent hyperinsulinism. In this large series, the phenotype ranged from isolated hemihypertrophy through to individuals with 7 additional major features of the disorder [5]. Our patient initially presented with isolated HH and, as a consequence, was screened for mutations in the K-ATP channel genes. The identification of a mosaic *KCNJ11* mutation(s) within the leukocyte DNA prompted us to undertake microsatellite analysis, which identified a loss of the maternal 11p15 allele within the pancreas and leukocytes, consistent with paternal UPD and a diagnosis of BWS. The genetic diagnosis of BWS was clinically confirmed later by the observation of hemihypertrophy of the right arm when the patient was 8 weeks of age.

## Conclusion

These results suggest that BWS should be considered in patients with newly diagnosed hyperinsulinaemic hyperglycaemia even in the absence of additional clinical features. In addition, UPD should also be considered in all patients with persistent hyperinsulinism with a paternally inherited, recessively acting K-ATP channel mutation even in absence of focal pancreatic disease.
